# Protective Effects of Dietary Supplementation with a Combination of Nutrients in a Transgenic Mouse Model of Alzheimer’s Disease

**DOI:** 10.1371/journal.pone.0143135

**Published:** 2015-11-25

**Authors:** Shengyuan Wang, Yu Cu, Chao Wang, Wei Xie, Lan Ma, Jinfeng Zhu, Yan Zhang, Rui Dang, Decai Wang, Yonghui Wu, Qunhong Wu

**Affiliations:** 1 Department of Social Medicine, Public Health College, Harbin Medical University, Harbin, China; 2 Department of Nutrition, YanZhou people's hospital, YanZhou, China; 3 Department of Nutrition and Food Hygiene, Public Health College, Harbin Medical University, Harbin, China; 4 Department of Occupational Health, Public Health College, Harbin Medical University, Harbin, China; CAEBi, SPAIN

## Abstract

**Objective:**

This study investigated the effects of intervention with a combination of nutrients in the amyloid precursor protein-presenilin (APP-PSN) C57BL/6J double transgenic mouse model of Alzheimer’s disease (AD).

**Methods:**

A total of 72 2-month-old APP-PSN mice were randomly assigned to three groups. The model group (MG) was fed regular, unsupplemented chow, while the low- and high-dose treatment groups (LG and HG, respectively) were given a combination of nutrients that included phosphatidylserine, blueberry extracts, docosahexaenoic acid, and eicosapentaenoic acid as part of their diet. An additional 24 wild-type littermates that were fed unsupplemented chow served as the negative control group (NG). After 3 and 7 months of treatment, the cognitive performance was assessed with the Morris water maze and the shuttle box escape/avoidance task, and the biochemical parameters and oxidative stress were evaluated in both the blood and brain.

**Results:**

An improvement in antioxidant capacity was observed in the treatment groups relative to the MG at 3 months, while superior behavioral test results were observed in the mice of the HG and NG groups. In the MG, pycnosis was detected in neuronal nuclei, and a loss of neurons was observed in the cerebral cortex and the hippocampus. At 7 months, the β-amyloid_1–42_ peptide accumulation was significantly elevated in the MG but was markedly lower in the mice fed the nutrient combination. The antioxidant capacity and behavioral test scores were also higher in these mice.

**Conclusions:**

Early intervention with a combination of nutrients should be considered as a strategy for preventing cognitive decline and other symptoms associated with AD.

## Introduction

Alzheimer’s disease (AD) is increasingly becoming a global public health burden with an estimated prevalence of over 100 million individuals by 2050 [1] because of the longer lifespan. As the most common form of dementia, AD is a progressive neurodegenerative disorder that is characterized by the presence of extracellular neuritic plaques and intracellular neurofibrillary tangles. The clinical symptoms of AD include memory loss, impaired cognitive function, a decline in the ability to perform day-to-day activities, and changes in personality and behavior [2, 3]. Although the precise etiology of AD is not fully known, it likely involves several processes, such as oxidative stress, abnormal processing of β-amyloid, epigenetic mechanisms, low levels of acetylcholine (ACh), mitochondrial dysfunction, and membrane deterioration, among others [4–9].

At present, there are not any effective therapeutic agents for treating AD [10, 11]. Furthermore, there are significant barriers to treatment, ranging from the physiological (the side effects of currently available drugs) to the economical (high fees for medical services and nursing care) factors [12]. Thus, new approaches for delaying the onset and progression of AD are urgently needed. With this goal in mind, early intervention with a combination of nutrients shows promise. More specifically, studies have reported that a variety of nutrients are beneficial for AD prevention, including B vitamins [13], lutein [14], lecithin [15], and omega-3 polyunsaturated fatty acids [16, 17], such as docosahexaenoic acid (DHA) and eicoapentaenoic acid (EPA). For instance, DHA can reduce Aβ accumulation as well as participates in neural repair and protection [16], and antioxidants reduce ROS-induced neuronal damage and stabilize membranes [18]. Furthermore, PS can improve cognitive function due to its numerous functions, including the regulation of receptors, enzymes, ion channels, and signaling molecules [19, 20]. Recently, the nutritional interventions study in humans suggested that supplementation with the macular carotenoids has the benefits on the patients with AD because of the improvements of visual function [21], while oral supplementation with long-chain polyunsaturated fatty acids or lutein/zeaxanthin had no statistically significant effect on cognitive function [22].

**Fig 1:**
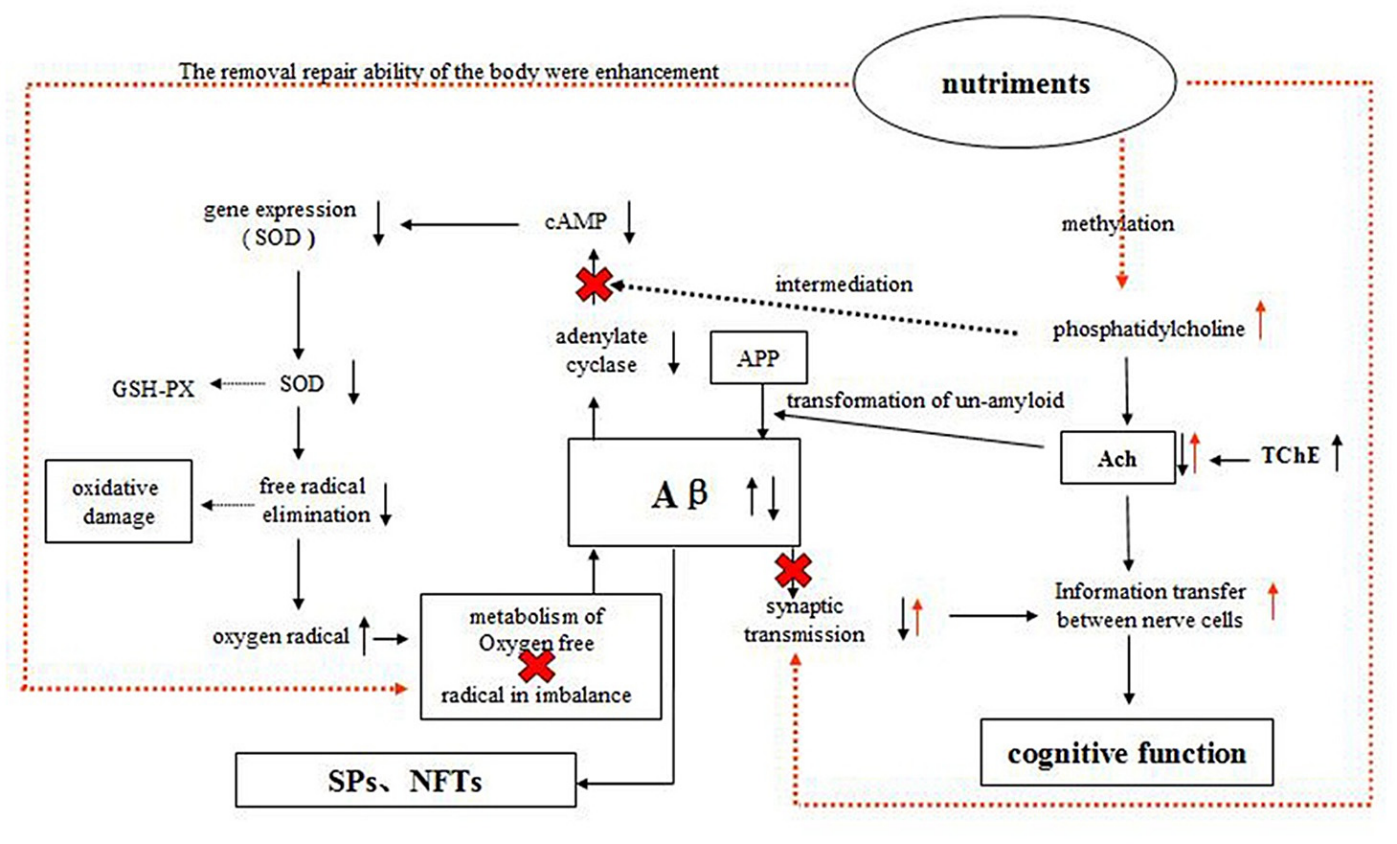
The possible mechanisms of action of compound nutrients. Ach: acetylcholine; cAMP: Cycilic adenosine monophospate; APP: amyloid precursor protein-presenilin; Aβ: anti-β-amyloid; GSH-PX: glutathione peroxidase; SOD: superoxide dismutase; TChE: total cholinesterase.

In addition, some nutrients, such as selenium, zinc, vitamin E, and blueberry extract, may provide a wide range of antioxidant benefits in AD [23, 24]. Subash et al. reported that the chronic dietary supplementation of date palm fruits showed possible beneficial effects concomitant with oxidative stress reduction, increased antioxidant enzymes, improved memory, learning and reduced beta amyloid in AD transgenic mice model [25, 26]. In addition, as part of the cell membrane, PS, EPA, and DHA directly modulate the activities of intracellular signaling and metabolic pathways that have a broad range of downstream physiological effects [27]. However, single-nutrient and -target study designs are somewhat limited [28], and the importance of considering an assortment of nutrients rather than just individual components is gaining increasing recognition in nutritional intervention studies [29]. The hypothesis of this design to mechanism of actionis shown in Fig 1.

In this sudy, the aim is to investigate the effect of dietary supplementation with phosphatidylserine (PS), blueberry extracts, DHA, and EPA on the cognitive function and oxidative stress in an amyloid precursor protein-presenilin (APP-PSN) double transgenic mice. The results demonstrated that the AD symptoms of animals receiving dietary supplementation were significantly diminished and oxidative stress were improved, which suggested that the incorporation of these ingredients into a diet could lead to protect against the cognitive deficits and cellular damage in the onset and the development of AD.

## Materials and Methods

### Materials

The Morris water maze, SDT-8 platform, and STT-100 shuttle box device were provided by the Cheng Du Taimeng Technology Co., Ltd. (Sichuan, China). The ingredients for the regular chow (corn starch, casein, maltodextrin, sucrose, powdered cellulose, and l-cysteine) were obtained from the Star Biological Technology Co., Ltd. (Beijing, China). The ingredients for the treatment (selenium; zinc; vitamins E, B12, and B6; folic acid; lutein; PS; DHA; and EPA) were purchased from the Beijing Boxing Biological Technology Co., Ltd. (Beijing, China). Blueberry extracts (≥ 50% procyanidine) were from the Daxinganling Lingonberry Organic Foodstuffs Co., Ltd. (Shanghai, China), and the anti-β-amyloid (Aβ)_1–42_ antibody (ab10148) was obtained from Abcam (Hong Kong).

### Animals

All experiments were performed in strict accordance with the recommendations found in the Guide for the Care and Use of Laboratory Animals of the Institute of Zoology. Furthermore, the study protocol was approved by the Medical Ethics Committee of Harbin Medical University. Amyloid precursor protein-presenilin (APP-PSN) C57BL/6J double transgenic mice (n = 72) and their wild-type littermates (n = 24) were purchased from the Model Animal Resource Platform of Nanjing University (license number SCXK [Su] 2010–0001). The transgenic mice express a chimeric mouse/human APP (Mo/HuAPP695swe) and mutant human PS (PS1-dE9) in CNS neurons under the control of independent mouse prion protein promoter elements associated with early onset AD [30, 31], and they develop Aβ deposits in the brain by 6 to 7 months of age [32]. Genotypes were confirmed by PCR genotyping using genomic DNA extracted from tail tissue samples, and the process was performed by the Model Animal Resource Platform of Nanjing University.

### Diet

The animals (2 months old) were acclimatized for 1 week, and then, the transgenic mice were randomly divided into three groups of 24 animals each, with equal numbers of males and females: the model group (MG) that was fed normal chow and the low and high treatment groups (LG and HG, respectively). Wild-type mice were fed normal chow and served as the negative control group (NG). The animals were maintained in a pathogen-free environment on a 12:12-h light/dark cycle with free access to food and water. The composition of ingredients in the nutrient combination diet is shown in Table 1 [33]. The high dose corresponded to the highest recommended intake for humans, while the low dose was half that of the high dose. The feed was prepared as granules, and both the food intake and body weight were recorded weekly for each animal.

**Table 1:** Feed formula and amount of intervention dose.

Component	MG/NG	LG	HG
zinc carbonate	57.75 mg/kg	115.50mg/kg	231mg/kg
sodium selenate	0.35 mg/kg	0.70mg/kg	1.4mg/kg
50%VE	150.00mg/kg	2.25g/kg	4.5g/kg
VB6	7.00 mg/kg	35.00mg/kg	70mg/kg
0.1%VB12	25.00 mg/kg	125.00mg/kg	250mg/kg
folic acid	2.00mg/kg	2.50 mg/kg	5mg/kg
lutein	——	1.00mg/kg.bw	2.00 mg/kg.bw
DHA+EPA (5:1)	——	100.00 mg/kg.bw	200.00 mg/kg.bw
phosphatidylserine	——	20.00 mg/kg.bw	40.00 mg/kg.bw
blueberry extracts	——	50.00 mg/kg.bw	100.00 mg/kg.bw

**Note**: (1) LG-low intervention group, HG- high intervention group, MG- model group, NG- negative control group. (2) Other components of the feed formula were based on AIN-93M. (3) The purity of each constituent was based on AIN-93M.

### Morris water maze

At 3 and 7 months after the start of the treatment regimen, 12 mice were randomly selected from each of the four groups for behavioral testing. Spatial learning and memory were assessed with the Morris water maze [34, 35]. The test consisted of 5 days of place learning and 1 day of space exploration in a black circular pool (diameter × height, 120 × 40 cm), with antholeucin (Harbin Sunshine Food Additive Co., Ltd., Harbin, China) added to the water (20°C ± 1°C) to render it opaque and the platform invisible. The pool was divided into four quadrants (I–IV) and was surrounded by black curtains. Landmarks placed inside the enclosure were used by the animal to locate the platform (7 cm diameter and 1.5 cm below the water surface) that was placed in quadrant III. Before the experiment, the animals were placed in a pool without the platform for 60 s to allow adaptation, and then, they were gently released from a starting point in one of the quadrants. Mice that found the platform within 60 s were permitted to stay on the platform for 10 s; the others were placed on the platform for 10 s. The place learning task consisted of four trial sessions per day for 5 days, and on the sixth day, the platform was removed from the maze, and the mice performed a 60-s probe trial test. A computerized tracking system was used to record and analyze the mice’s movement.

### Shutter box escape/avoidance task

The experiment was carried out in shutter boxes consisting of two equal-sized compartments (25 × 25 × 28 cm) connected by an opening (8 × 10 cm) [36]. Diffused illumination was provided by a fluorescent bulb that was placed in the ceiling of the boxes. Mice were acclimatized inside the shutter box shock area for 5 min and were then given the conditioned stimulus (lights) for 20 s, followed by the unconditioned stimulus (a 24-V, 50 Hz electric shock though the grid floor) for 10 s. One round of the task was complete when the animal crossed to the other compartment, with crossing during the conditioned or unconditioned stimulation being considered as avoidance and escape responses, respectively. The training consisted of a single 40-trial session over the course of 1 day, with the test repeated after 24 h. A camera was placed in the center of the ceiling in each box t so aso record and analyze the mice’s movement.

### Biochemical parameters

A total of 12 mice in each group were used for the behavioral studies after 3 and 7 months of treatment. The mice fasted for 12 h; then, they were anesthetized with 10% chloral hydrate, and a blood sample was drawn from the eyeball for biochemical analyses. The brain was removed from six of the animals in each group, frozen in liquid nitrogen, cut into two halves by midsagittal dissection, and then kept at −80°C. The left half was ground using a mortar and pestle on dry ice and afterwards was homogenized in normal saline (1:9 weight in g:volume in ml). The homogenate was centrifuged at 4°C and 2500 × *g* for 15 min, and the supernatant was used to measure the levels of superoxide dismutase (SOD), glutathione peroxidase (GSH-Px), malondialdehyde (MDA), total cholinesterase (TChE), and total protein by spectrophotometry (except for MDA) (8500 UV-VIS; Techcomp, Ltd., Shanghai, China) using the appropriate kit (Nanjing Jiancheng Biochemistry Co., Nanjing, China) according to the manufacturer’s protocols. The only deviation was a dilution of the supernatant by 10-fold with normal saline for the SOD and protein measurements. The blood was centrifuged at 20°C and 3000 × *g* for 15 min. The serum was stored at 4°C for a maximum of 3 days before its use in assays for MDA, SOD, acetylcholine (ACh), and TChE.

MDA was assayed with the thiobarbituric acid heating method. To measure the SOD activity, the method employing the reaction of superoxide radicals with hydroxylamine to form a red formazan dye at 550 nm was used. The SOD activity was then measured as the degree of inhibition of this reaction, and the results were given as U/mg or U/ml in the brain and serum, respectively. The GSH-PX activity was measured by the consumption of reduced glutathione in the enzymatic reaction with 5,5'-dithiobis(2-nitrobenzoic acid), which resulted in a yellow solution at 412 nm. TChE hydrolyzes ACh, generating acetic acid and choline, which can react with a thiol chromogenic agent to form the yellow compound 1,3,5-trinitrobenzene at 412 nm. The protein content in the brain was quantified using the Coomassie brilliant blue assay.

### Histology and immunohistochemistry

Six mice per group, mice that were not used for the brain biochemistry assays, were transcardially perfused with 25 ml of ice-cold saline containing 30IU heparin sodium followed by 50 ml of 4% paraformaldehyde in 0.01 M phosphate-buffered saline (pH 7.2–7.4). Their brains were removed and post-fixed in the same solution overnight, and then, they were embedded in paraffin and sectioned at a thickness of 4 μm. The resulting sections were placed overnight in an oven at 68°C, then deparaffinized in three changes of xylol, rehydrated in a graded series of alcohol, and washed with distilled water. They were then immersed in fresh 3% H_2_O_2_ to inactivate endogenous peroxidase and were again washed with distilled water. For antigen retrieval, the sections were placed in 10 mmol citrate buffer (pH 6.0) for 4 min at 2100°C in a pressure cooker. Afterwards, they were cooled to room temperature over 40 min and then blocked in 5% bovine serum albumin and incubated with a primary antibody against Aβ_1–42_ overnight at 4°C. The sections were incubated with biotinylated anti-mouse IgG secondary antibody for 20 min and then with a diaminobenzidine (DAB) solution using the Vector DAB peroxidase substrate kit (Vector Laboratories, Burlingame, CA, USA), followed by counterstaining with methylene blue for 20 s. After clearing in xylene, the sections were cover-slipped for examination under a light microscope. Some sections were stained with hematoxylin and eosin (H & E) for histopathological examination [37].

### Data analysis

Statistical analyses were performed with SPSS 17.0 for Windows (SPSS Inc., Chicago, IL, USA). Data corresponding to biochemical parameters were presented as mean ± standard error of the mean and were analyzed with the unpaired student’s t test. The results from the Morris water maze and avoidance task were analyzed by analysis of variance (ANOVA) for repeated measures and the χ^2^ test, respectively. P < 0.05 was considered statistically significant.

## Results

### Animal body weight and food intake

**Fig 2:**
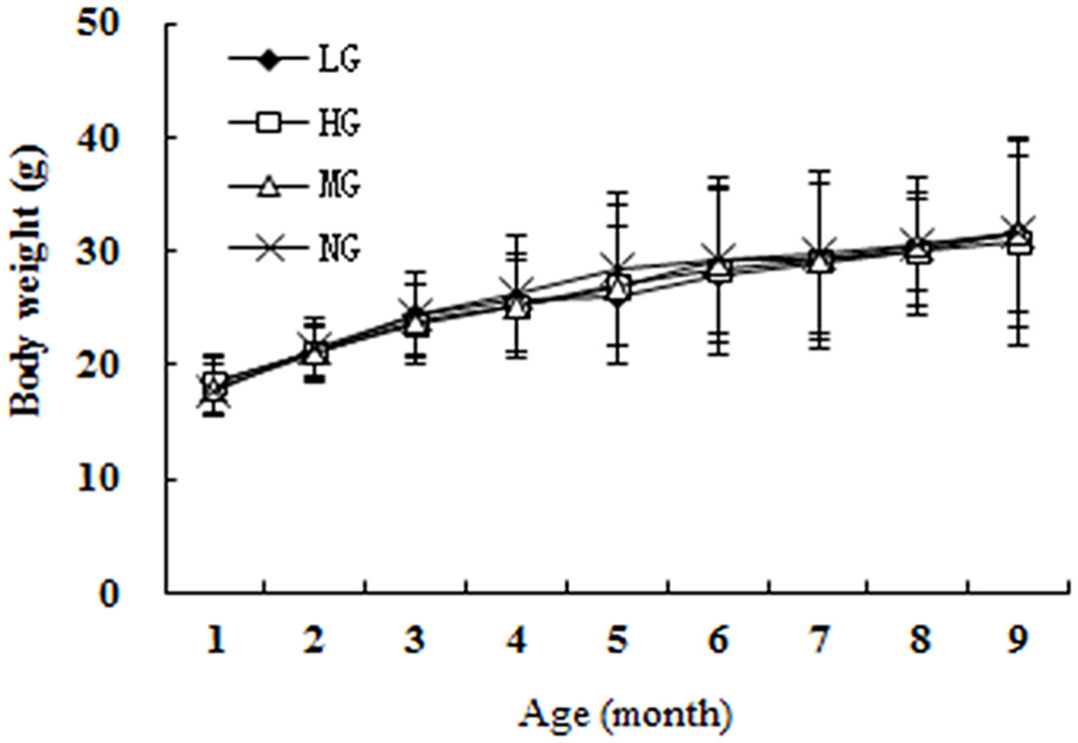
The trend of the average weights of the four groups.

There was no significant difference in the daily food intake across groups (LG, 3.39 ± 0.18 g; HG, 3.34 ± 0.22 g; MG, 3.63 ± 0.26 g; NG, 3.51 ± 0.23 g), and over time, the body weights of all the mice (regardless of group affiliation) were similar up to 9 months of age (Fig 2).

### Behavioral tests

**Fig 3:**
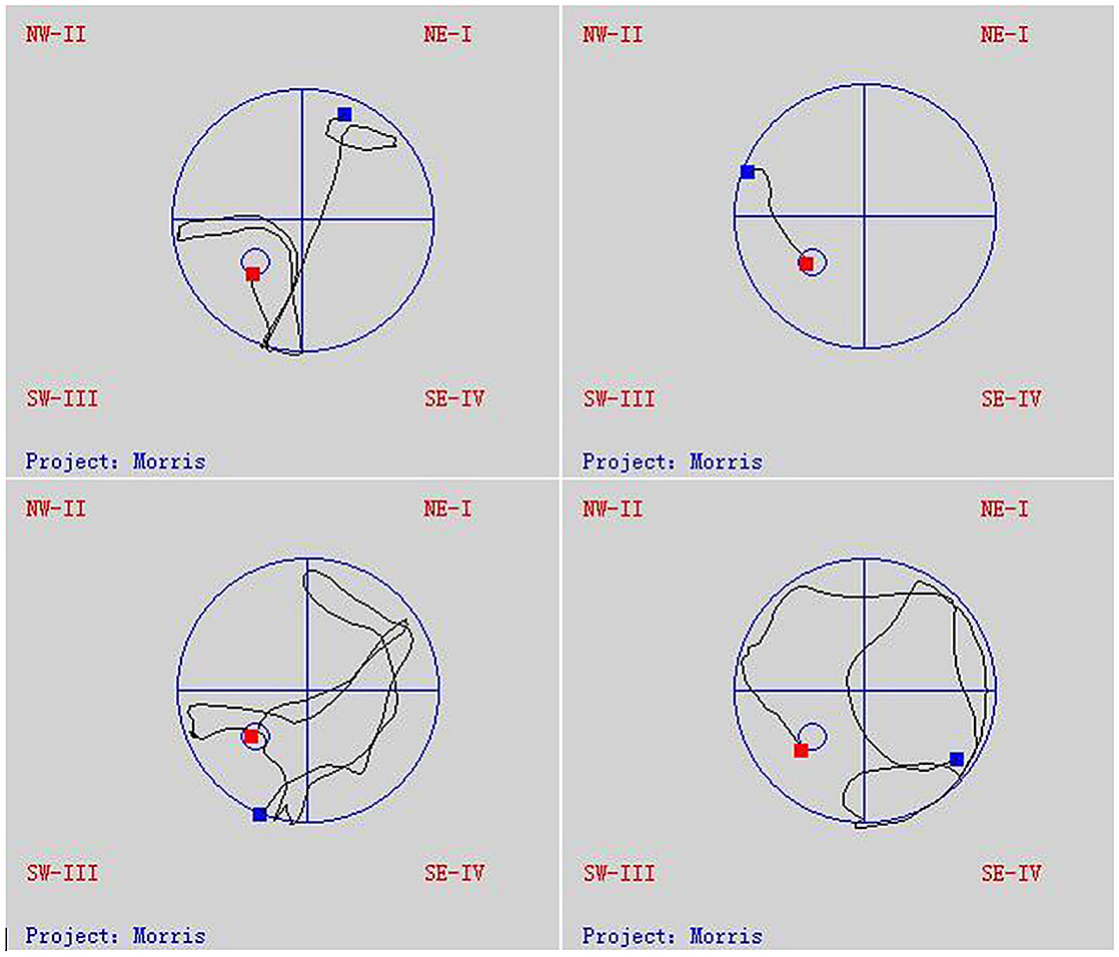
Trajectories of mice in the Morris water maze tests.

**Fig 4:**
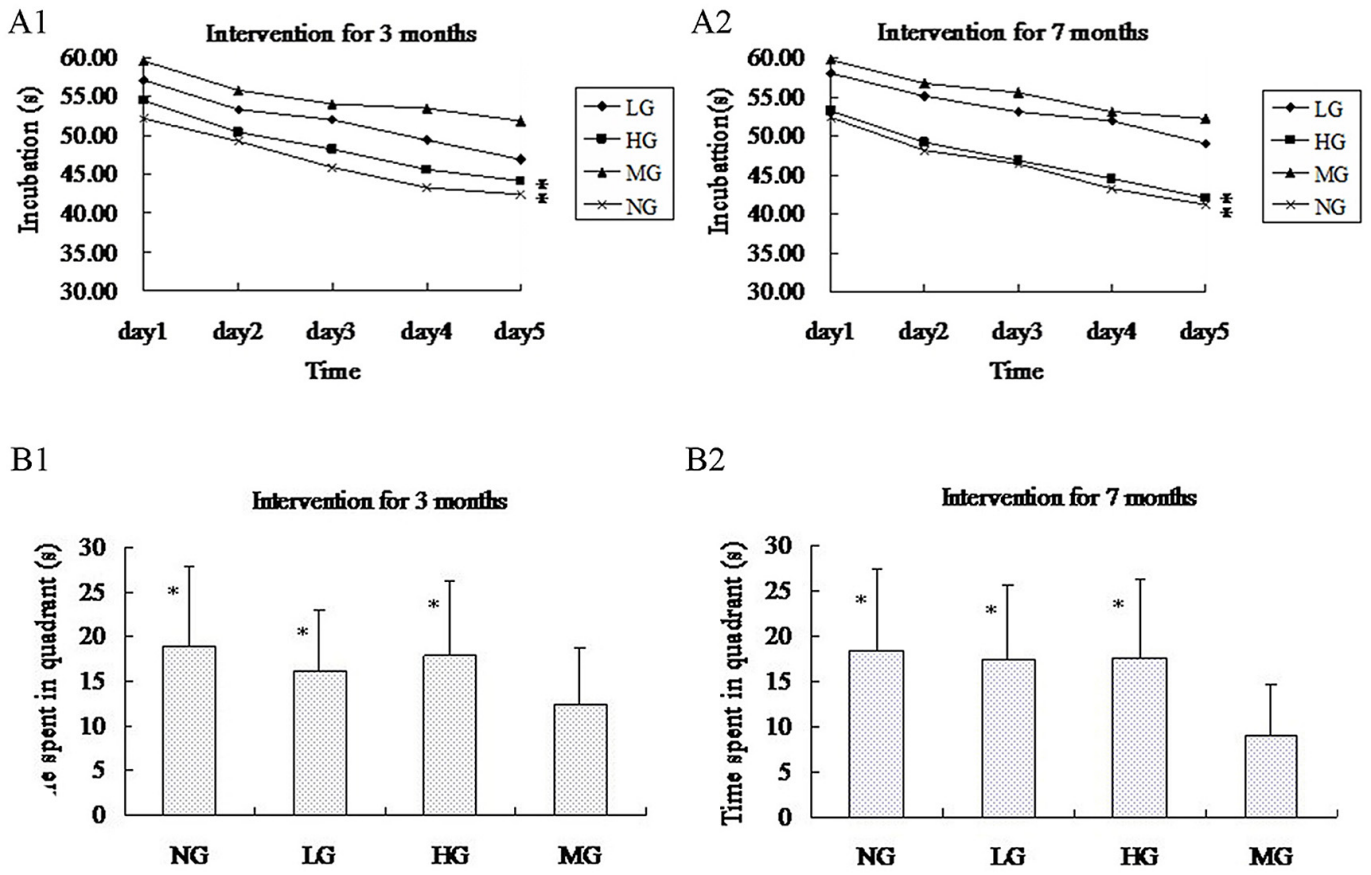
Results from Morris water maze tests. (A) The average incubation period from the first to fifth day for the four groups. After 3 months and 7 months of intervention, the mice in HG and NG spent much less time searching for new (reversal) hidden platform on each reversal training day compared to MG, * P<0.001. (B) The time spent in the third quadrant for HG, LG, and NG was much greater than that of the mice in MG, * P<0.01.

The Morris water maze test took place over 5 days, and the mice were provided different entry points to the pool (Fig 3). The latency until the mice found the platform in each trial became shorter over this period, indicating that the mice were learning to navigate the maze (Fig 4A). At 3 months after the start of treatment, the NG and HG had shorter latencies than the MG (repeated measures ANOVA [38]; P < 0.001), but the same was not true for the LG (P = 0.310). Likewise, at 7 months, only the NG and HG (P < 0.001), but not the LG (P = 0.972), performed better on the test than the MG. There was no statistical difference in the latency between the NG and HG (at 3 months, P = 0.596; at 7 months, P = 0.972). Similarly, the time spent in quadrant III on the sixth day after the removal of the platform was shorter in the NG and HG than in the MG (unpaired student’s t test; P < 0.01 for both groups at 3 and 7 months), while the LG did not differ from the MG (at 3 month, P = 0.106 and at 7 months, P = 0.203) (Fig 4B).

In the shutter box escape/avoidance test, the percentage of active or passive avoidances and escape failures during the 40-trial session was recorded (Table 2). Compared to the MG, there was a higher incidence of active avoidance and fewer escape failures in the NG, HG, and LG at 3 and 7 months, (P < 0.001 for all groups at both time points). The escape latency was also lower in the NG and HG than in the MG (unpaired student’s t test; at 3 and 7 months, P < 0.01 for both groups).

**Table 2:** Results from the two-way (shuttle-box) active avoidance training.

	LG***	HG***	MG	NG***
Intervention for 3 months				
Active avoidances response(%)	45.00	46.67	20.83	44.38
Passive avoidances response(%)	30.42	27.92	40.63	28.96
Escape failures(%)	24.58	25.41	38.54	26.66
Escape latency(s)	6.90±2.29*	5.75±2.58*	9.58±2.85	5.15±1.52*
Intervention for 7 months				
Active avoidances response(%)	30.63	45.00	29.38	42.29
Passive avoidances response(%)	53.33	25.63	21.46	32.92
Escape failures(%)	16.04	29.38	49.17	24.79
Escape latency(s)	7.60±2.57*	7.33±2.05*	10.58±3.00	6.92±1.67*

**Note:** (1) LG-low intervention group, HG- high intervention group, MG- model group, NG- negative control group. (2) The percentage of active avoidances, passive avoidances, and escape failures *** P<0.001, compared with MG. (3) Escape latency(s): the average response latency for the 40-trials in the shuttle box training session; n = 12, * *P*<0.01.

### ACh and TChE levels in serum and brain

The ACh serum level was similar across all groups at 3 months (Table 3); however, at 7 months, a higher level was observed in the HG and LG compared to the MG (P < 0.05). Conversely, the serum TChE level was reduced in the HG, LG, and NG compared to the MG at both 3 and 7 months (P < 0.05).

**Table 3:** Distribution of Ach and TChE in serum and brains for the four groups (Mean ± SD, n = 6/12).

	LG		HG		MG		NG	
	3 m	7 m	3 m	7 m	3 m	7 m	3 m	7 m
In Brain								
Ach (μg/mg port)	56.43±12.03	56.80±17.10*	74.81±9.45**	53.11±12.59**	50.74±11.65	35.08±3.44	70.50±10.01**	58.28±8.96***
TChE (U/mg port)	0.91±0.29	0.84±0.37	0.83±0.18*	0.73±0.24*	1.14±0.29	0.99±0.15	0.79±0.17*	0.74±0.16*
In serum								
Ach (μg/ml)	282.54±123.42	305.95±98.88	321.82±120.41	380.65±115.32*	294.18±96.33	269.64±108.59	355.53±141.71	431.84±175.96*
TChE (U/ml)	47.31±3.51**	59.75±7.56*	46.82±3.08**	53.23±5.54***	52.60±5.48	66.40±5.55	46.83±3.99**	58.17±6.13**

**Note:** (1) LG-low intervention group, HG- high intervention group, MG- model group, NG- negative control group. (2) *: *P*<0.05, **: *P*<0.01, *** *P*<0.001 compared with MG. (3) in brain, n = 6; in serum, n = 12.

In the brain, ACh was increased in only the HG at 3 months and in the HG, LG, and NG at 7 months (Table 3). In contrast, the TChE levels were lower in the HG, LG, and NG than in the MG at both 3 and 7 months.

### Indicators of oxidative stress

The MDA level in the serum at 3 months was lower in both the LG (P < 0.05) and HG (P < 0.01) than in the MG (Table 4); moreover, the level in the HG was markedly lower than in the LG (P < 0.05). In contrast, the SOD level was higher in the NG, HG, and LG compared to the MG at 3 months. Similar trends were also observed at 7 months.

**Table 4:** Distribution of indicators of oxidative stress of brain and serum for the four groups (Mean ± SD, n = 6/12).

	LG		HG		MG		NG	
	3 m	7 m	3 m	7 m	3 m	7 m	3 m	7 m
In Brain								
Total Protein (g/l)	0.40±1.13	0.47±0.22*	0.36±0.06*	0.41±0.05***	0.50±0.10	0.68±0.08	0.34±0.05**	0.39±0.06***
SOD (U/mg port)	3.09±0.39*	2.41±0.36*	3.18±0.64*	2.72±0.42**	2.25±0.76	1.94±0.33	3.08±0.76*	2.47±0.43*
MDA (nmol/mg)	2.30±0.66*	2.82±0.92*	2.23±0.95*	2.83±0.89*	3.73±1.15	4.62±1.65	2.06±0.85*	2.74±0.72*
GSH-PX (U/mg port)	26.03±14.90	30.51±9.15*	41.12±5.71***	31.45±1.39***	22.44±4.35	20.55±1.36	41.59±6.49***	31.10±2.03***
In serum								
MDA (n mol/ml)	6.89±2.64*	9.15±3.47*	6.71±1.75**	7.36±2.06***	8.71±1.19	12.04±1.28	6.43±1.68***	9.41±3.30*
SOD (U/ml)	129.16±37.28**	123.26±28.567*	140.17±30.85***	129.90±19.80**	96.05±11.79	86.06±43.53	139.80±37.28***	131.91±25.49**

**Note:** (1) LG-low intervention group, HG- high intervention group, MG- model group, NG- negative control group. (2) *: *P*<0.05, **: *P*<0.01, *** *P*<0.001 compared with MG. (3) in brain, n = 6; in serum, n = 12.

The MDA, SOD, and GSH-Px levels in the brain tissue are shown in Table 4. Relative to the levels in the MG, the MDA level was lower in the NG, HG, and LG, whereas the SOD and GSH-Px levels were higher. Furthermore, the total protein content in the brain was lower in these three groups than in the MG at 7 months (P < 0.05).

### Pathologic changes in brains

**Fig 5:**
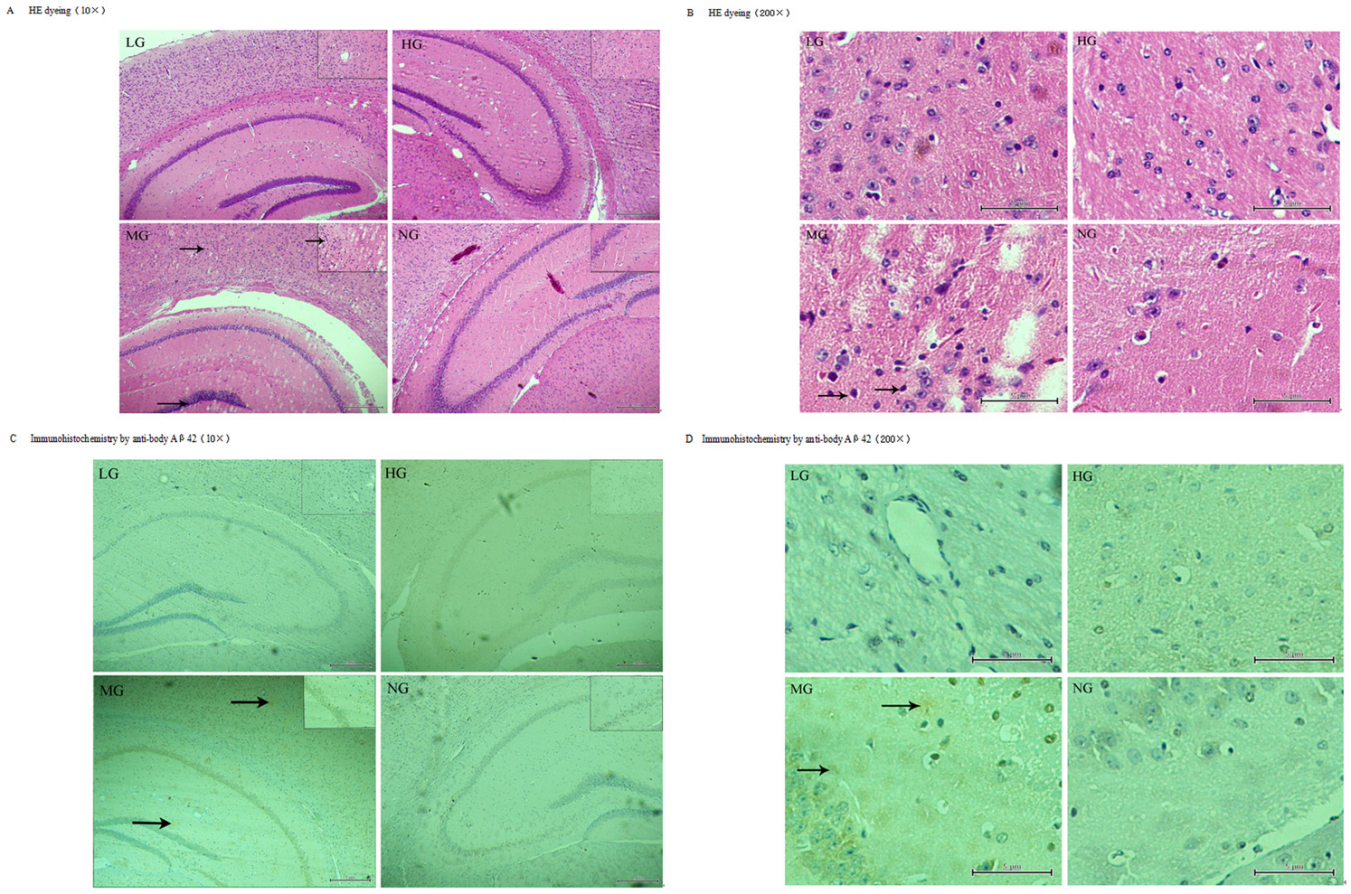
Immunofluorescence staining with rabbit anti-Aβ1–42 and HE dyeing of the temporal cortex and hippocampus of APP/PSN mice. (1) LG and HG: APP/PSN (Fig 5A) mice with 7 months of intervention; MG: APP/PSN (Fig 5B) mice without intervention and their wild-type littermates (NG). (2) LG and HG: ±, MG: +++, NG:–(Fig 5C and 5D).

At 9 months old, fewer neurons and pycnotic neuronal nuclei were observed in the cerebral cortex and hippocampus of the transgenic mice but not the wild-type by H & E staining (Fig 5A and 5B). Immunohistochemistry with an antibody against Aβ_1–42_ (Fig 5C and 5D) revealed an increase in the number and volume of amyloid plaques proportional to the age of the mice (data not shown). Additionally, fewer plaques were observed in the cortex and hippocampus of the mice in the LG and HG after 7 months of treatment compared to the MG at the same age (Fig 5D), thus suggesting that early intervention with this nutrient combination can suppress the development of amyloid plaques.

## Discussion

In this study, it was demonstrated that APP-PSN mice experience a significant improvement in cognitive function as well as in biochemical and histological indicators of AD after consuming a mixture of nutrients for up to 7 months.

Cognitive dysfunction is one of the major clinical manifestations of AD and is correlated with the loss of hippocampal and cortical synapses [39] that serve as the cellular substrates for spatial learning and memory [35]. In this study, the rate of memory decline was reduced along with the amelioration in cognitive function in mice after dietary intervention, thereby suggesting that this could be a possible strategy for delaying or preventing AD symptoms in humans [40].

During aging, oxidative stress resulting from reactive oxygen species (ROS) production increases even as endogenous antioxidant mechanisms progressively decay. These could be exacerbated in AD. In fact, a growing body of evidence implicates the early involvement of oxidative stress in the pathogenesis and progression of AD [41, 42]. The attack of lipids, proteins, sugars, and nucleic acids by free radicals leads to the formation of byproducts that can be detected in fluids and tissues and ultimately can serve as markers of oxidative damage [43]. Accordingly, lower levels of the oxidative stress indicators MDA, SOD, and GSH-PX were observed in the mice that were receiving the dietary supplement in this study, which could be due to the antioxidant micronutrients, such as selenium, lutein, zinc, and vitamin E.

Acetylcholinesterase and the cholinergic system, which are involved in learning and memory, may also play a role in AD. TChE belongs to a family of α/β hydrolases that catalyze the hydrolysis of ACh at the cholinergic synapse [44]. Some studies have described a decrease in ACh and an increase in the TChE level in the brain as contributing factors to memory dysfunction in AD [45–47]. The present findings were consistent with these reports. The AD mice consuming the nutrient mixture had a higher ACh level than those who were fed regular chow, while the TChE level was reduced. Thus, increasing the ACh level in the brain by modulating TChE activity and signaling pathways mediated through cholinergic receptors that promote nonamyloidogenic APP processing and decrease tau phosphorylation are potential strategies for AD therapy [48]. Furthermore, the amyloid cascade hypothesis has been proposed to explain the pathogenesis of AD. The deposition of Aβ is the initial pathological event, which then triggers a cascade of events that include the formation of senile plaques, followed by neurofibrillary tangles, and this cascade ultimately results in neuronal death and dementia [49, 50]. Here, it was found that the Aβ deposition in the treatment groups was decreased compared to the AD model mice who were fed a regular diet. Thus, the protective effects of these nutrients may be direct—by promoting neural repair and inhibiting the formation of amyloid plaques—or else auxiliary, through antioxidative mechanisms or by lowering homocysteine.

Lastly, there are some limitations to this study. First, it is not clear that the beneficial effects of the tested nutrient combination would translate to humans. Moreover, not all of the components are commercially available. In the future, preclinical trials will be carried out to assess specific early biomarkers of AD using metabolomics approaches. In conclusion, the findings presented here suggest that a combination of nutrients may synergize to delay the symptoms of AD and that early intervention by dietary supplementation is a viable strategy for AD management.
