# Correction: Protective Effects of Dietary Supplementation with a Combination of Nutrients in a Transgenic Mouse Model of Alzheimer's Disease

**DOI:** 10.1371/journal.pone.0146252

**Published:** 2015-12-29

**Authors:** Shengyuan Wang, Yu Cui, Chao Wang, Wei Xie, Lan Ma, Jinfeng Zhu, Yan Zhang, Rui Dang, Decai Wang, Yonghui Wu, Qunhong Wu

The second author’s name is spelled incorrectly. The correct name is Yu Cui. The correct citation is: Wang S, Cui Y, Wang C, Xie W, Ma L, Zhu J, et al. (2015) Protective Effects of Dietary Supplementation with a Combination of Nutrients in a Transgenic Mouse Model of Alzheimer’s Disease. PLoS ONE 10(11): e0143135. doi:10.1371/journal.pone.0143135
